# Miss-A-Nail Technique for Neck of Femur Screw Fixation: It Is Easier Said Than Done

**DOI:** 10.5704/MOJ.2003.017

**Published:** 2020-03

**Authors:** RY Kow, A Abdul-Aziz, CL Low

**Affiliations:** 1 Department of Orthopaedic, Hospital Tengku Ampuan, Afzan (HTAA), Kuantan, Malaysia; 2 Department of Orthopaedic, International Islamic, University Malaysia (IIUM), Kuantan, Malaysia; 3 Department of Radiology, Hospital Tengku Ampuan Afzan, (HTAA), Kuantan, Malaysia

Dear editor,

Antegrade nailing is considered the “gold standard” in treating femoral shaft fractures^1^. Peri-implant fractures occurring at the femoral neck in young adults are uncommon. Femoral neck fractures occurring in young adults are often due to high-energy trauma^1^. Treatment of femoral neck fractures are often performed with open or closed reduction with cannulated or non-cannulated screw fixation^1^. With an antegrade femoral nail in situ, it complicates the neck of femur fixation. One option includes removing the previous implant prior to inserting the screws for the neck of femur fractures. However, removal of the implant may cause iatrogenic fractures. The removal may not be feasible due to the overgrowth of bone on top of the nail and locking screws insertion sites^1^. Any attempt to remove the previous implant with invasive maneuvers risks further displacement of the neck of femur fracture and increases the risk of avascular necrosis of the femoral head. Miss-A-Nail technique can be performed for femoral neck screw fixation with the femoral nail in situ^2^.

**Fig. 1:**
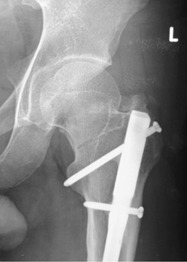
Plain radiograph of a 26-year-old man who sustained an undisplaced neck of femur fracture in a motor-vehicle accident 6 years after the greater trochanter (GT) entry femoral interlocking nail has been inserted.

**Fig. 2:**
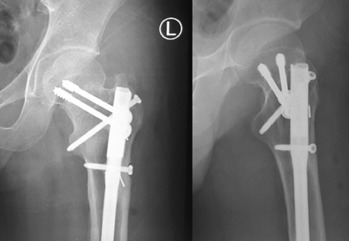
Plain radiographs of the patient after the Miss-A-Nail technique screw fixation for the femoral neck fracture. Note that the remnants of the broken 2.0mm threaded guide wire are not retrieved as they are embedded inside the femoral bone.

**Fig. 3:**
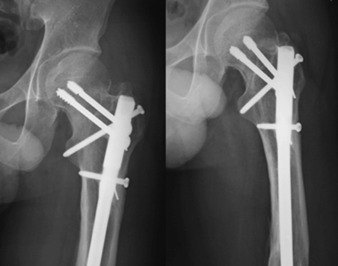
Plain radiographs of the patient shows radiological union at one month after the Miss-A-Nail technique screw fixation for the femoral neck fracture. The patient is able to return to full activity at two months after the surgery.

A 2.0mm threaded guide wire is normally inserted at 100, 110, 120, or 130 degree, placing either anterior or posterior to the previous femoral nail^2^. A 7.0mm stainless steel cannulated screw is inserted at the path of the guide wire after drilling with a 4.5mm cannulated drill bit. We found that during insertion of the 2.0mm threaded guide wire, it is inevitable that the guide wire will hit either the path of the nail or proximal locking screws due to the limited spaces available. Furthermore, a worn-out guide wire will easily break at the thread runout once it hit the path of nail or proximal screw. In order to overcome this problem, we replace the threaded guide wire with a 2.5mm Kirschner wire to negotiate a path away from the nail towards the femoral head without breaking it when it hit the path of the nail or proximal locking screw. The 2.5mm Kirschner wire is then removed and the 2.0mm threaded guide wire is inserted into the path created by the 2.5mm K wire. We also utilise the washer at the end of each 7.0mm cannulated screw to prevent the cannulated screw from penetrating the hip joint as the proximal femur bone quality may be suboptimal due to the load sharing devices (femoral nail) in situ. Depending on the trajectory of the previous proximal locking screw(s), one may need to insert the screw at a less-than-100 degree to avoid the previous locking screw(s). In our center, a 26-year-old man with an undisplaced neck of femur fracture underwent the femoral neck screw fixation via the described Miss-A-Nail technique and he subsequently recovered and returned to work four months after the surgery (Fig. 1-3)
